# Human Bocavirus DNA in Paranasal Sinus Mucosa

**DOI:** 10.3201/eid1708.101944

**Published:** 2011-08

**Authors:** Valeria Falcone, Gerd J. Ridder, Marcus Panning, Sibylle Bierbaum, Dieter Neumann-Haefelin, Daniela Huzly

**Affiliations:** Author affiliation: Freiburg University Medical Center, Freiburg, Germany

**Keywords:** viruses, human bocavirus, paranasal sinus mucosa, multiplex real-time PCR, letter

**To the Editor**: Human bocavirus (HBoV) is a newly described parvovirus for which pathogenic potential has not clearly been elucidated (*1*). Recent findings suggest that HBoV may establish persistent infection of mucosal lymphocytes or contribute to tonsillar hyperplasia in children (*2*). In previous reports, we described prolonged HBoV DNA detection in immunocompromised children (*3**,**4*). Partial sequencing of the VP1 gene of HBoV from bronchoalveolar lavage fluid, plasma, and sphenoid sinus samples showed 100% identity, which suggested persistence of the same HBoV strain over a 5-month period (*3*). It remains speculative, however, whether paranasal sinus mucosa represents a site of HBoV persistence. To clarify this, we analyzed samples of paranasal mucosal tissue and nasal polyps from patients with chronic sinusitis for respiratory viruses and atypical bacteria.

A total of 102 tissue samples were obtained from 88 patients (median age 48.5 years, range 13.3–88.1 years) from July 2009 through September 2010 after elective surgery. Indication for surgery was established by otorhinolaryngologists. The most common indication was chronic sinusitis. No patients displayed acute respiratory symptoms at the time of surgery. To detect asymptomatic shedding in the upper respiratory tract and viremia, we collected nasal swabs and EDTA-blood samples concurrently. The study protocol was approved by the Ethics Committee of the University of Freiburg. Informed written consent was obtained from all study participants.

Approximately 25 mg of each tissue specimen was used for nucleic acid extraction by using an RNeasy Mini Kit, as described (*5*) (QIAGEN, Hamburg, Germany). To provide evidence that the QIAGEN RNeasy kit is also suitable for DNA extraction, we spiked HBoV negative samples with different amounts of HBoV DNA before extraction of nucleic acids was done with either the QIAGEN RNeasy Kit or DNA Blood Kit (QIAGEN). Extracted nucleic acids were then subjected to real-time PCR by using primers specific for HBoV. Minimal differences (±1 cycle threshold [C_t_] value) in the HBoV PCR were detected; the QIAGEN RNeasy Kit was therefore used throughout the study (data not shown). Nasal swabs and ETDA-blood were purified by using a QIAamp MinElute Virus Spin Kit (QIAGEN). Multiplex PCR for respiratory viruses (Fast-track Diagnostics, Junglinster, Luxembourg) was conducted to detect influenza A (including pandemic [H1N1] 2009) and B viruses; respiratory syncytial virus; human metapneumovirus; HBoV; parainfluenza virus 1–4; human coronaviruses HKU1, NL63, 229E, and OC43; human rhinoviruses; human enteroviruses and parechoviruses; and adenoviruses. *Mycoplasma pneumoniae, Chlamydia pneumoniae, Legionella pneumophila*, and *Bordetella pertussis* were analyzed as described (*6**–**8*).

A single virus was detected in 22/102 (21.5%) tissue specimens, with HBoV being the most frequent (18/102, 17.6%), followed by rhinovirus (2/102, 1.9%), coronavirus 229E, and influenza A pandemic (H1N1) 2009 virus (1/102, 0.9%). HBoV was detected in specimens collected during July–September (13/18) and during February and March (5/18). All positive results were confirmed by single real-time PCR (*9*). For 14 patients, 2 different mucosal samples were tested and gave identical results. No multiple viral infections and no bacteria were detected. Median patient age was 51.2 years (range 14.4–74.2 years) for HBoV-positive and 47.6 years (range 13.3–88.1 years) for HBoV-negative samples. C_t_ analysis in single real-time PCR revealed a median C_t_ of 31 (range 28–38), corresponding to 200 genome equivalents/10^6^ cells (range 3–1.8 × 10^4^ copies/10^6^ cells). No correlation between C_t_ value and patients’ age was observed (r^2^ = 0.008; data not shown). No underlying disease was diagnosed for 13/18 HBoV-positive patients, whereas 2/18 and 3/18 patients had chronic obstructive pulmonary and oncologic disease, respectively.

Nasal swabs and EDTA-blood samples were obtained from 17/18 and 7/18 HBoV-positive patients, respectively. No HBoV was detected in any swabs or EDTA-blood samples available, indicating no virus shedding in the respiratory tract and no viremia. However, the 2 patients with rhinovirus-positive samples obtained from biopsy also had rhinovirus RNA detectable in nasal swabs, suggesting rhinovirus infection. Unfortunately, no nasal swab was available from the 2 patients whose sinus biopsy samples were positive for HCoV 229E and influenza A virus.

In this study, we simultaneously analyzed tissue specimens of paranasal sinuses and nasal polyps, as well as nasal swabs and blood samples, for a broad panel of viruses and atypical bacteria. To avoid seasonal bias, specimens were collected over a 1-year period and exclusively obtained from patients undergoing elective surgery in the absence of acute respiratory symptoms.

The finding that HBoV was present as a single virus in 18/22 virus-positive biopsy samples is intriguing. Moreover, the fact that no HBoV DNA was detected in nasal swabs or EDTA-blood samples indicates no active HBoV infection. In previous studies, HBoV DNA was frequently identified in the adenoids and tonsils of children (*2**,**5**,**10*). However, in contrast with our findings, detection of HBoV was mostly associated with other viruses, suggesting that co-virus–induced cellular damage might contribute to bocavirus reactivation and replication (*5*). Our findings indicate that persistence of viral nucleic acid in sinus mucosa might be a special advantage of HBoV, although the relevance of this observation remains unclear. Whether this presence as a single virus means a dead end for HBoV infection, true latency including the potential of reactivation, or a role in the pathogenesis of clinical conditions requiring surgery warrants future studies.
